# Positive impact on vitamin D related lifestyle of medical advice in pregnant Somali-born women and new mothers: a mixed method study in Swedish primary care

**DOI:** 10.1186/s12889-021-10277-y

**Published:** 2021-02-05

**Authors:** P. Kalliokoski, M. Widarsson, N. Rodhe, M. Löfvander

**Affiliations:** 1 Department of Public Health and Caring Sciences, Family Medicine and Preventive Medicine Section, Uppsala University, Husargatan 3, Postal address: Box 564, 751 22 Uppsala, Sweden; 2 School of Health, Care and Social Welfare, Mälardalen University, Västerås, Sweden; 3 Centre for Clinical Research, Uppsala University, Västerås, Sweden

**Keywords:** Mixed method, Lifestyle, Prevention, Primary care, Public health, Somalia, Transcultural consultation, Vitamin D, Women

## Abstract

**Background:**

A previous study showed that pregnant women/new mothers especially Somali-born and some Swedish-born had extremely low vitamin D levels and poor physical performance. Our study aimed to examine vitamin D related lifestyle, attitudes and behaviour before and after brief information about vitamin D, with special long-term focus on Somali-born women.

**Methods:**

A cohort of 91 pregnant women/new mothers having serum hydroxyvitamin D (S-25-OHD) ≤ 50 nmol/L (*n* = 51 Somali-born with one third < 10 nmol/L of S-25-OHD) in primary health care in Sweden was targeted for intervention. Brief individual oral and visual information on vitamin D was given by doctors at baseline and after four and ten months. Questionnaires with ordinal scales on vitamin D related lifestyle of food, clothing, and outdoor activities were distributed on all occasions. Focus-group interviews with 15 women from the target-group were performed after two years. A Somali interpreter was available.

**Results:**

Veiled clothing, indoor living, and a low intake of milk, cheese, and fatty fish were common in the target group. Consumption pattern had increased significantly among the Somali-born women at the four-month follow-up but declined to non-significant levels at the ten-month follow-up. The focus-group interviews showed improved understanding of vitamin D deficiency, symptoms and attitudes, but varying applied behaviours related to sun exposure. Sun exposure for the children and increased fish consumption was the most evident positive results.

**Conclusions:**

Vitamin D related lifestyle, attitudes and behaviour improved in a Somali-born group of pregnant women/new mothers with severe vitamin D deficiency. The preventive measures suggested in our study may have impact on public health in relation to bone and muscle strength and immunity especially in vitamin D deficiency risk groups.

**Trial registration:**

ClinicalTrials.gov Identifier: NCT02922803. Date of registration: 28 September 2016.

## Background

Large differences in vitamin D levels were documented between Somali-born and Swedish-born pregnant women and new mothers [1]. Lifestyle is crucial for adequate vitamin D levels. The importance of vitamin D for bone and muscle health has been known for a long time [2–5]. Vitamin D has raised international interest due to its tentative immuno-protective role against viral and bacterial infections [6, 7].

Vitamin D has been of certain interest in the Nordic and other countries far from the Equator [8–11]. Ultraviolet radiation (UV-light) for synthesising vitamin D_3_ in the skin is limited to a few months in summer in these regions [2, 12, 13]. For the remaining periods of the year, the main sources for vitamin D is food containing vitamin D like fatty fish and vitamin D_3_ enriched food like milk and margarine [14].

All Somali-born women in our first cross-sectional study from antenatal care in mid-Sweden, wore veiling clothes, were seemingly weak and had a feeble way of walking (Trendelenburg walk) and tended to fall when attempting to squat [1]. No Swedish-born pregnant/new mother failed in doing the physical performance tests although some of them had low vitamin D levels (c.f. flowchart). Nearly 90% of the Somali-born women had severe vitamin D deficiency, serum 25-hydroxyvitmin D (S-25-OHD) < 25 nmol/L. One third of them had immeasurable levels of S-25-OHD i.e. below ten nmol/L. Most of them also had elevated levels of parathyroid hormone and alkaline phosphatase indicating a risk for osteomalacia [1, 15]. These women thus needed immediate intervention because of the high demands of vitamin D and calcium during pregnancy and lactation [15–17]. Low health related to quality of life and low vitamin D concentrations are common among Somali-born women in Sweden [18]. Being main providers of food and childcare, any improved lifestyle changes to obtain more sun exposure and food for them would possibly also benefit their families, especially their children [19].

Several publications have a focus on food intake and vitamin D concentrations [20–23]. To the best of our knowledge, there are no previous publications including medical advice on vitamin D, lifestyle and food to pregnant women with severe vitamin D deficiency, regardless of country of birth, social circumstances or trans-cultural health care aspects.

With consideration to the above aspects, we designed a follow-up study regarding knowledge on vitamin D matters and lifestyle before and after Swedish doctor’s information for groups of pregnant women/new mothers of Somali and Swedish birth, with prominent vitamin D deficiency.

The main research question was if brief information and advice on vitamin D and related lifestyle, provided by doctors during ordinary consultations, have a noticeable impact on knowledge and behaviour associated with vitamin D.

The aim of this paper was three-fold: First, to study if vitamin D related lifestyle differed between women having normal and deficient vitamin D levels within groups of Somali-born and Swedish-born pregnant women/new mothers. Second, to explore changes in vitamin D related lifestyles within the Somali and Swedish groups with deficient vitamin D levels. Third, to explore knowledge, attitudes and behaviour related to vitamin D among the Somali-born participants after 2 years.

We hypothesised that the target group of Somali-born women on long-term, had reached an awareness of vitamin D and vitamin D related lifestyle and that this had resulted in modified attitudes and behaviour.

## Methods

### Design and setting

A before and after treatment study using a mixed method design. The setting was an antenatal and primary healthcare clinic in the town of Borlänge in mid-Sweden at latitude 60^o^ N. The recruitment period and a baseline study was performed in May to June 2010. Follow-ups took place after four and ten months and ended in April 2011. A two-year follow-up with focus group interviews was performed in May 2012.

### Participants

**Fig. 1:**
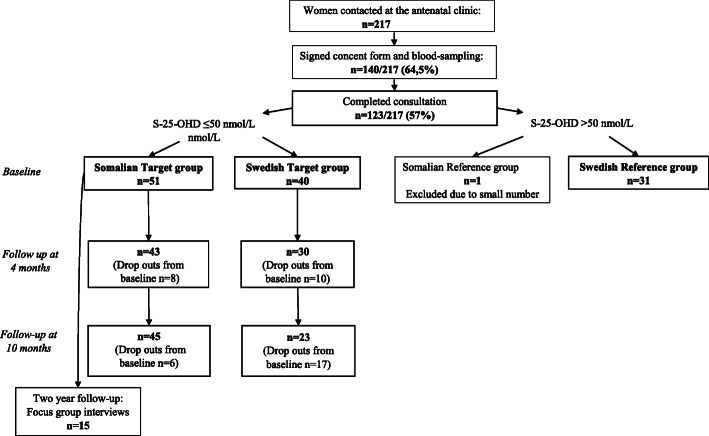
Flow chart showing participating women born in Somalia and Sweden.

A total of 217 women enlisted at the antenatal clinic were contacted. One hundred and twenty-three women (57% of the 217) completed the baseline consultation of which 52 were Somali born, see flowchart Fig. 1. All women having S-25-OHD ≤ 50 nmol/L constituted the target study group for intervention. They were categorised into a Somali target group (SomTG) and a Swedish target group (SweTG). All women with S-25-OHD > 50 nmol/L were referred to as a reference group divided into a Swedish group (SweRG) and a Somali group (SomRG). All the 52 Somali-born women from baseline were eligible for the focus group interviews. Fifteen women came for the focus group interviews after two years.

### Programme at a glance

Muscular performance, questionnaires and vitamin D were studied at baseline in springtime for all the 123 participants. They received the same vitamin D related advice from one of the two research doctors.

The follow-ups focused on the participants with S-25-OHD ≤ 50 nmol/L. Vitamin D_3_ supplements containing calcium (800 IU or 20 μg cholecalciferol and 500 mg calcium carbonate) were prescribed to all the 91 participants for the whole study period. Two tablets daily were prescribed to participants with S-25-OHD < 25 nmol/L and one tablet daily for those with 25–50 nmol/L. Follow-ups at four months, in autumn, and  ten months, later after winter, included the same tests and lifestyle questions. All Somali-born women from baseline were invited to the focus group interviews two years later. A Somali-born female interpreter was available for the Somali women and reminded them of the scheduled appointments. The research doctors contacted the Swedish-born women.

Two-thirds of the Somali-born and one-third of the Swedish-born women had given birth (new mothers) at baseline.

### Data collection at baseline and follow-ups

Serum vitamin D (S-25-OHD) concentration was measured using a Liaison 25 OH Vitamin D total assay (DiaSorin, Stillwater, MN, USA) in a certified clinical chemistry laboratory at University Hospital, Uppsala, Sweden. The method consistently had 10–20% lower values than the specific LC-MS reference methods used at other laboratories [24]. The method did not require fasting, but participants had fasted for a minimum of 8 h (from midnight of the previous day to the time of testing) since fasting parathyroid hormone also was measured. Samples were centrifuged before transport, kept cool and secured from UV-light. The total CV (intra- and inter CV) of the method was 2,3% at 42,9 nmol/l and 1,97% at 145 nmol/l. Grip strength was measured with hand dynamometer in Newton (Grippit) [25]. Ability to perform squats, hip lift (Trendelenburg), stand up and one leg stand was rated but not used in this paper.

### Questionnaires for baseline and follow-ups

Questionnaires for personal data and lifestyle associated with vitamin D, social- and anthropometric data were completed at all occasions. Vitamin D related lifestyle questions were the following:

- Approximately how many decilitres or glasses of milk, sour milk or yoghurt do you drink/eat every day? Answers in decilitres, or glasses.
- How often do you eat cheese? Answers were ticked in boxes: never (= 0), 1–2 times/w (= 2), 3–5 times/w (= 4) and 6–7 times/w (= 6). Figures within brackets are for the calculations.
- How many times per week do you eat salmon or other fatty fish? Never (= 0), 1–2 times (= 1), 3 times or more (= 3).
- Are you outdoors at least 30 min every day in summer? No (= 0) or yes (= 1).
- Do you expose face, neck and forearms to sun during summer? No (= 0) or yes (= 1).
- Do you sunbathe in a sunny country 1 week or more during winter? No (0) or yes (1).

### Intervention

Information on vitamin D was adapted for persons with little education. No written material was distributed. A Somali nurse and professional interpreter were available. Oral information on how vitamin D is produced in the skin and why it is important was presented in a straightforward language. Basic visual material was shown comparing the angles of sun light and the distance through the atmosphere in Sweden during summer and winter and to light at the equator (Somalia). Food containing vitamin D was shown in a picture. This was communicated in consultation with either one of the two doctors (male and female). All participants were given the same information regarding the skin as the main source of vitamin D production via ultraviolet light from the sun. In addition, information was given about food containing Vitamin D. The emphasis was on fatty fish, cheese – as an important companion to vitamin D for its content of calcium, and on egg yolk and certain mushrooms. Particular emphasis was on low and middle fat milk and light margarines because these products are enriched with vitamin D in Sweden and have been for a long time [14].

Emphasis was also on the efficacy of exposure of the face, neck and forearms to the sun, and that the more skin exposed areas mean increased vitamin D production. The Somali-born women were informed that darkly pigmented skin shields from sunburns and cancer but takes longer time to produce vitamin D. All women were informed that clothing covering the body blocks production of vitamin D and that winter sun is insufficient because sunrays travel long distances through the atmosphere. This was illustrated by a figure of the sun radiation at the equator, and in Sweden, during summer- and wintertime. Another figure showed vitamin D rich food. Adequate vitamin D levels and normal calcium absorption was explained in brief. The main symptoms emanating from longstanding very low blood vitamin D levels were described mentioned as ‘bone softening’ (e.g., osteomalacia).

### Follow-ups at four and ten months

All SweTG and SomTG women were invited for the follow-ups with new S-25-OHD measurements, lifestyle questionnaires and muscular performance tests.

### Follow-up at two years

Only the 52 Somali-born women were the focus for group interviews on vitamin D related issues. These interviews were performed in order to identify the participants’ current understanding, attitudes and behaviour towards vitamin D. The interpreter nurse invited the women to the primary health care centre by phone and those who accepted the invitations were further invited to participate in focus group interviews conducted by a female Swedish-speaking anthropologist. The previous interpreter translated all Swedish sentences into Somali. Sixteen women consented to participate, nine of them came to the first interview and six women for the next.

The first and the last author together with the interviewer prepared the interview guide containing thematic questions in order to enlighten the emic perspectives of the women’s attitudes towards lifestyle regarding vitamin D (food, sun exposure, sunbathing and clothing) and health. The interviews lasted one and a half hours each. The content was recorded and transcribed word by word by the interviewer. The transcriptions were repeatedly read by the first, second and last authors and subsequently analysed using systematic text condensation according to Malterud [26]. Meaning units of words in the text covering food, sun, symptom, and vitamin D were highlighted in different colours and summarised into themes. The themes were de-contextualised into categories by using meaningful units. The designated categories were compared with the interviews from which appropriate quotations were selected and presented in the results.

### Statistics

Numbers, percentages, mean and median values with standard deviation and inter-quartile ranges were calculated for lifestyle data associated with vitamin D and for S-25-OHD, socio-demographic and anthropometric data.

Comparisons of vitamin D related lifestyle data at baseline between the SweRG and the two target groups were calculated using the t-test (interval values) and the Mann-Whitney Test for ordinal scales and the chi-square test for nominal data.

Vitamin D related lifestyle and food consumption data at baseline and at the follow-ups were compared using the Wilcoxon signed-rank (ordinal data) and the McNemar’s four-field test (categorical data).

A *p*-value < 0.05 was considered statistically significant.

Calculations were done using IBM SPSS Statistics version 23.

## Results

One hundred and forty women out of two hundred and seventeen agreed to participate in the study and came for blood sampling and the written consent (140/217, 64.5%), 58 of whom were Somali-born and 82 Swedish-born. They received an appointment ten days later to perform physical tests, fill in questionnaires and to receive information. One hundred and twenty-three women (52 Somali, 71 Swedish) came to the baseline study, see flowchart Fig. 1. Of the 140 women who agreed to participate in the study, two gave birth in between the occasions and did not turn up and the remaining 17 did not offer reasons for dropping out.

The target group population constituted of the 91 participants (51 Somali, 40 Swedish) who had S-25(OH)D ≤ 50 nmol/L. The remaining 32 women constituted the tentative reference group. Only one Somali woman had S-25(OH)D > 50 nmol/L and therefore no Somali reference group was presented. Life-style data at baseline on food containing vitamin D, cheese, sun exposure and veiling clothing was missing only for one Somali and one Swedish woman.

Different drop outs on the two follow-ups were observed, albeit few, between four- and ten-month follow-ups, as seen in flow chart, Fig. 1. The common reasons given by the respective 18 and 23 dropouts for not showing up, was that they either felt fine and/or did not want to participate any longer or was unknown. Among those who responded to the questionnaires at four and ten months only a few missing life-style values (*n* = 1–3) was seen.

Table 1 shows the baseline characteristics for the 52 Somali-born and 71 Swedish-born participants. Formal schooling was much higher among the Swedish women. Mean residential time in Sweden was 3.5 years for the Somali women. One-third (35%) of them had immeasurable levels (less than 10 nmol/L) of serum 25-OHD. Immeasurable values of S-25-OHD were replaced with a 9 in the statistical data calculations providing a mean of 16 nmol/L for Somali vs. 49 nmol/L for Swedish women. Only one Somali woman had ‘normal’ levels of S-25-OHD. The Somali women in general consumed little milk, cheese and fatty fish, spent limited time outdoors, had low sun exposure during summer and no sun vacation during winter. They usually wore veiled clothing including the traditional burka.

**Table 1:** Characteristics of the 123 pregnant women and new mothers at baseline

	Somali-born	Swedish-born
n	52	71
Age, years, m (SD)	28 (6)	31 (5)
Height, cm, m (SD)	161 (5)	167 (6)
Education, years, m (SD)	3.3 (3.4)	13.8 (2.5)
Years in Sweden, m (SD)^1^	3.5 (2.6)	
S-25-OHD, nmol/L, m (SD)^2^	16 (10)	49 (18)
Grip strength, N, m (SD)	199 (58)	318 (66)
Milk, dl/day, m (SD)	1.8 (2.0)	3.6 (1.9)
Cheese times/w, md (IQR)^3^	2.5 (2.5)	4.3 (1.7)
Fish times/w, md (IQR)^4^	0.7 (1.0)	0.8 (0.5)
Exercise times per week, md (IQR)^5^	1.5 (1.5)	1.8 (1.2)
Outdoors, n (%)^6^	31 (61)	66 (96)
Veiled clothing, md (IQR)^7^	1.1 (0.3)	0.0 (0.0)
Sun exposure face, neck, forearms, n (%)	13 (25)	65 (96)
Sun vacation in winter, n (%)	1 (2)	11 (16)

^1^ Most of the Swedish-born women had lived all their lives in Sweden

^2^ 1/3 of Somali-born women had 25-OHD < 10 nmol/L. The value < 10 was replaced with a 9 for the calculation of the mean. The Somali median value was 12 nmol/L

^3^ Cheese times/w; 0 = never, 2 = 1–2 times/w, 4 = 3–5 times/w, 6 = 6–7 times/w

^4^ Fish times/w; 0 = never, 1 = 1–2 times/w, 3 = 3 or more times/w

^5^ Exercise times per week; 0 = seldom or never, 1 = 1–2 times/w, 3 = minimum 3 times/w or more

^6^ Outside minimum 30 min per day in summer

^7^ Veiled clothing; 0 = short armed sleeves, short legs/skirts in summer, 1 = long sleeves/legs without covering face and hands, 2 = long arms/skirts, scarf covering head/face, burka

*n* Numbers, *m* Mean, *SD* Standard deviation, *cm* Centimeter, *N* Newton, *dl* Deciliter, *w* Week, *md* Median, *IQR* Interquartile range

Table 2 shows vitamin D lifestyle data for the reference group (SweRG *n* = 31) and the target groups (SweTG *n* = 40; SomTG *n* = 51). Milk consumption differed much between the SweTG and the SweRG women (3.1 vs 4.2 decilitre). The SomTG women consumed significantly less milk per day, only 1.8 dl/day. They had also significantly lower data for consumption of other food, and a less healthy lifestyle like outdoor and physical activities. Only Somali-born women used veiled clothing.

**Table 2:** Lifestyle data at baseline in the Swedish reference group (RG) with serum hydroxyvitamin D > 50 nmol/L and the Somali and Swedish target groups (TG) with serum hydroxyvitamin D ≤ 50 nmol/L.

	Swedish RG	Somali TG		Swedish TG	
			*p*		*p*
n	31	51		40	
Milk, dl/day, m (SD)	4.2 (1.7)	1.8 (2.0)	**< 0.001**	3.1 (1.9)	**0.021**
Cheese times/w, md (IQR)^1^	4.4 (1.2)	2.4 (2.5)	**0.001**	4.2 (2.0)	0.875
Fish times/w, md (IQR)^2^	0.9 (0.3)	0.7 (1.0)	**0.005**	0.8 (0.6)	0.176
Outdoors, n (%)^3^	29 (97)	30 (60)	**< 0.001**	37 (95)	0.717
Veiled clothing, md (IQR)^4^	0.0 (0.0)	1.1 (0.3)	**< 0.001**	0.0 (0.0)	1.00
Sun exposure face, neck, forearms, n (%)	28 (97)	12 (24)	**< 0.001**	37 (95)	0.739
Sun vacation in winter, n (%)	5 (17)	1 (2)	**0.016**	6 (15)	0.885

*p*-values = Swedish RG compared with Somali and Swedish TG

*p*-values < 0.05 in bold calculated by t-test for milk deciliter/day; Mann-Whitney test for the ordinal scales cheese and fatty fish times/w and for degree of veiled clothing; chi-square test for answer yes for being outdoors during summer and sun exposure

^1^ Cheese times/w; 0 = never, 2 = 1–2 times/w, 4 = 3–5 times/w, 6 = 6–7 times/w

^2^ Fish times/w; 0 = never, 1 = 1–2 times/w, 3 = 3 or more times/w

^3^ Outdoor minimum 30 min per day in summer

^4^ Veiled clothing; 0 = short armed sleeves, short legs/skirts in summer, 1 = long sleeves/legs without covering face and hands, 2 = long arms/skirts, scarf covering head/face, burka

*n* Numbers, *dl* Deciliter, *m* Mean, *SD* Standard deviation, *w* Week, *md* Median, *IQR* Interquartile range

Table 3 shows the vitamin D related lifestyle data for the TGs at baseline, and four and ten months later. The drop-out rate was low in the SomTG, and 43% in the SweTG.

**Table 3:** Lifestyle data at baseline and after four and ten months in the Somali and Swedish target groups (TG) with baseline serum hydroxyvitamin D ≤ 50 nmol/L

	Somali TG					Swedish TG				
	Baseline	4 months	*p*	10 months	*p*	Baseline	4 months	*p*	10 months	*p*
n^1^	50	43		45		39	30		23	
Milk, dl/day, m (Std)	1.8 (2.0)	3.0 (2.0)	**0.001**	2.6 (2.1)	0.066	3.1 (1.9)	3.4 (1.9)	0.497	3.5 (3.8)	0.972
Cheese/w, md (IQR)^2^	2.4 (2.5)	3.7 (2.3)	**0.006**	3.8 (2.4)	**0.009**	4.2 (2.0)	4.3 (1.7)	0.129	4.2 (2.1)	0.415
Fish/w, md (IQR)^3^	0.7 (1.0)	1.3 (1.1)	**0.027**	1.2 (1.0)	0.095	0.8 (0.6)	0.9 (0.6)	0.317	0.9 (0.6)	0.317
Outdoor, n (%)^4^	30 (60)	33 (77)	0.118	–	–	37 (95)	28 (97)	1.000	–	–
Veiled clothing (0–2), md (IQR)^5^	1.1 (0.3)	1.0 (0.5)	0.285	–	–	0.0 (0.0)	0.03 (0.2)	0.317	–	–
Sun exposure of face, neck, forearms, n (%)	12 (24)	17 (40)	0.092	–	–	37 (95)	27 (93)	1.000	–	–
Sun vacation in winter, n (%)	1 (2)	–	–	2 (4)	1.00	6 (15)	–	–	0 (0)	0.250

*p*-values by Wilcoxon signed rank test for paired samples for the categories drinking milk, eating fat fish, exercise and veiling clothing

*p*-values by Related Samples Mc Nemar test for the categories outside (more than 30 min/day), sun exposure (face, neck, forearms) and sun vacation abroad (minimum one week per winter)

^1^ Missing values (*n* = 1–3) in each category are not reported in the table

^2^ Cheese times/w; 0 = never, 2 = 1–2 times/w, 4 = 3–5 times/w, 6 = 6–7 times/w

^3^ Fish times/w; 0 = never, 1 = 1–2 times/w, 3 = 3 or more times/w

^4^ Outside minimum 30 min per day in summer

^5^ Veiled clothing; 0 = short sleeves, legs/skirts in summer, 1 = long sleeves/legs without covering face and hands, 2 = long sleeves/skirts, scarf covering head/face and burka

- no data for this variable at ten months follow up

*n* Numbers, *dl* Deciliter, *w* Week, *m* Mean, *SD* Standard deviation, *md* Median, *IQR* Interquartile range

At the four-month follow-up, the SomTG women reported significant increase in consumption of daily milk, cheese and fatty fish. However, at the ten month follow-up, these decreased to statistically non-significant higher values compared with baseline. A trend toward more sun exposure during summer was observed at the four-month follow-up (*p* = 0.09). There was no change in veiled clothing. The SweTG women reported no changes in food consumption or lifestyle habits.

The S-25-OHD levels after intervention are not shown because of impacts from the possible consumption of vitamin D supplements. Lifestyle changes on the S-25-OHD levels could thus not be evaluated.

### Two-year follow-up with focus group interviews – Somali participants

Results from the focus group interviews with Somali-born women indicated that they had obtained an understanding of vitamin D. Majority of the participants had increased their daily intake of vitamin D rich foods, and cheese, and so had their family members, especially the children. Most of the interviewed women displayed a new knowledge compared to the previous non-existent knowledge of vitamin D, its content in certain foods and the importance of sun exposure for vitamin D. However, there were diverging attitudes towards vitamin D related behaviour regarding to sun exposure but not when it came to food. Most of them tried hard to change their behaviour, both personally and when caring for their children especially for being active outdoors, expose skin to sun and serving foods rich in vitamin D and taking supplement.

The following four categories were identified from the analysis: Health and understanding of symptoms, Food and supplements, Strong Somalia sun - Weak Swedish sun, and Garments – hindrance for catching some sunrays. Appropriate quotes are presented under each theme.

### Health and understanding of symptoms

The essential part was that they felt worse now than at baseline or at the time of immigration. They felt more tired, weaker and had more leg pain. However, this could be due to older age, stress, as well as lack of sun and vitamin D:

‘*When we came, we were young’ ‘…We are older now. ‘…I was stronger before I came, and weaker and weaker when in Sweden’.* Some, however, did not agree: ‘*I have no worse health, but I lack vitamin D because I have no sun here’.*

Several mentioned mental symptoms like exhaustion, nervousness, irritation, vertigo, insomnia due to lack of vitamin D.

‘…*never felt like this in Somalia’* One woman was nervous about wasting of skeleton…’ ‘*Is it true that the skeleton vanishes when you lack vitamin D?*’

### Food and supplements

Food was easier to accept than lack of sun.

‘ …*We eat the food, but miss the sun’!*

They now had positive attitudes towards food containing vitamin D – milk, margarine and fish like salmon and cheese containing calcium compared with previous life ‘.*… we ate small sharks in Somalia’.* But some were worried about all the food ‘…*We watch TV about putting on weight because much food’.* Some experienced improved health of vitamin D medication *‘… I feel weak in my legs if I don’t take them’.* Others felt side effects like palpitations, trembling, weariness, and dizziness. Still, some of them continued and experienced that side effects subsided and eventually ceased altogether*…You get used to side effects’… ‘they vanish’.* Yet some stopped taking the medication even if perceiving good effects*. ‘…became better, but I did not continue. I don’t know why* ‘. It seemed however that most relied on vitamin D rich foods instead of supplements: ‘…*I’ve heard that egg and salmon help, so I try to eat the food instead of the tablets.*

### Strong Somali sun - weak Swedish sun

A new insight for most of the women was that the sun was a source of vitamin D but doubted if the Swedish sun was good*:*

*‘We had sun in Somalia and vitamin by the sun’*. ‘*We walk to catch sun, but we are scared… sun can be dangerous; we will get sick and become weak’. ‘…sun in Somalia didn’t give you headache like the sun here…’ ‘if too much sun here – maybe you get cancer?’ or ‘I will get heart failure from the sun here’* or the Swedish sun could have other side effects *‘…When I sit in the sun, I become dizzy…’.*

Despite doubts about the Swedish sun, they, nowadays, as a rule, tried to expose their skin to more sunshine: *‘…if sun, I go outdoors’.* This changed behaviour included the children. ‘…*we walk in the forest and sunbathe with the children...’ ‘I like sun, I use to turn my face to the sun’.* One woman had learned of the benefits of the sun and now removes her veil when outdoors. Others were not as direct but said they liked to sunbathe. Many said they try to go outside every day with the children because they know it gives them vitamin D.

### Garments – hindrance for catching sun rays?

The women were unanimous in saying that sun behaviour differed between Somali-born and Swedish-born women because a Somali woman needs a proper place for catching the rays lest unknown men spot her. ‘…*We don’t walk outdoors as much, I don’t take off my clothes, except a little when on the balcony’* because *‘Everyone shall have clothes on from the age of 15’.* But opinions and behaviour could differ *‘…it depends on how religious you are’.* Other barriers for lack of sun exposure and veiled clothing were illness and Islamic law. Such comments were few but existed: ‘*According to Islamic law, she cannot do so…*’. ‘…‘*I must obey the law even when sick’ ‘You have to check that no man is there’.*

## Discussion

The target group of Somali women reported a significantly increased intake of fatty fish, cheese and milk at the four-month follow-up. This change was less evident after ten months. They also had a trend towards increased sun exposure of skin but not in time spent outdoors or less use of veiled clothing. At two-year follow-up focus-group interviews confirmed a positive change in attitudes and behaviour also including their children and a novel awareness of disorders from vitamin D deficiency.

The results of our study should be interpreted with caution. For example, the self-reported nature of data is a limitation due to recall or reporting bias. Another limitation is the language barriers and contextual understanding despite the use of a professional translator. Furthermore, focus group interviews include group influences that possibly may affect wording and content when talking about attitudes and behaviour. There is also a risk for bias in the selection of participants in strategic sampling. However, this was possibly avoided because all of the enlisted Somali-born women were contacted at the antenatal care and statistically randomised Swedish-born women randomised by parity.

Somali migrants are common in Europe and cross-cultural studies in Britain showed that the Somalis living in Liverpool often had bone and muscle pain and had food intake reflecting traditional customs, with a low content of vitamin D and calcium and little milk consumption [23]. Our Somali participants had comparatively a higher intake of milk at baseline, which had increased considerably at the follow-ups.

A Finnish study found differences in intake of healthy foods, where the Somali women had a lower fish consumption than the Russian female immigrants who in turn did not differ much from Finnish women [27]. In our study the Somali-born women at baseline had much lower intake of milk, cheese and fatty fish especially as compared with the Swedish reference group with much higher vitamin D levels.

Our study showed new insights and increased awareness regarding lifestyle, sun and food among our Somali-born women with little education and with very low, or severely low, vitamin D levels. This positive result could possibly be linked to the brief oral and visual information given via interpreter. Such intervention, minus interpreter, had seemingly no or only slight impact on the comparatively well-educated group of SweTG women with less severe vitamin D deficiency. Evaluation in this group is however hampered due to their varying numbers of drop-outs in the follow-ups. In addition, the Swedish-born participants had doctor encounters only while the Somali-born participants had doctor encounters and contact with the nurse interpreter, which could have had an unknown impact on the results, and the comparatively lower drop-out rate.

Scandinavian transcultural intervention studies are few. A Norwegian study of new mothers from Middle-East and from Somalia found no change in vitamin D status between the intervention group having had written information, illustrations and advice and the controls having had general oral information on health [28]. Another study with 151 persons including Somali participants, had significantly improved their Healthy Eating after 12 months compared with controls, with a persistent improvement also at one year [29].

A Finnish study showed differences in the need for oral vitamin D_3_ between Finnish non-pregnant women with 10 μg more vitamin D_3_ daily needed among Somalis to keep S-25(OH)D levels above 30 nmol/L [30]. In addition, the consumption of fresh vegetables, fruits and berries was particularly low among the Somali immigrants [27]. Similar results were found in Sweden among dark-skinned compared to fair-skinned children [31]. These findings may seem discouraging when compared to the modestly increased intake of vitamin D rich foods in our study where the increase of two glasses of vitamin D enriched milk per day would contribute to a rise of four extra micrograms of vitamin D_3_. But any improved lifestyle, attitudes and behaviours regarding vitamin D will improve S-25(OH)D levels and health especially for women immigrating to higher latitudes [8]. Intake of vitamin D rich food is extra important also for persons with malabsorption, e.g., coeliac disease, or for vegans abstaining from meat and milk products and thereby increase their risk for osteomalacia [32, 33]. Dark-skinned persons and women using traditional veiled clothing are other risk groups for osteomalacia but seem protected for skin cancer [19, 34].

Positive results on improved lifestyle was found from a mixed method study evaluating a five-week long culturally tailored health-promotion program provided by clinicians and a local coordinator for Arabic and Somali-speaking women [19]. A qualitative study from US, however, showed that newly immigrated parents use strategies to preserve cultural and religious values in order to strengthen the Somali community [35]. That one, and other qualitative studies, indicate how difficult it can be for some immigrant groups to change to new habits regarding clothing, food and lifestyle [36, 37]. Our modest, but encouraging, results should be viewed in that context.

A Swedish primary care study found that most immigrant women with veiled clothing had low concentrations of 25(OH)D, and that sun vacation, enriched food and supplements were insufficient in avoiding this [20]. Notably, 15 min daily of exposure (uncovered skin, Fitzpatrick Skin type II or III) in midday sun during summer time can yield sufficient vitamin D levels [38]. However, at latitudes above 60°N, vitamin D from skin production is only possible during April to September with a peak at noon but the magnitude of this contribution is unknown [39].

Despite limitations, we believe and hope that the positive results from our study can be applicable and possible to transfer to similar migrant populations of women elsewhere. However, transferability can be limited by the time elapse, societal change and context, and also be influenced by ongoing acculturation and social context. The benefits are the applied real-life design as a clinical study including oral and visual information, interpreter support, blood sampling and physical tests by doctors in an ordinary consultation surrounding. In addition, there were rather few drop-outs among the newly immigrated pregnant women with few years of formal education and very low vitamin D levels. In contrast, there were many drop-outs among the Swedish-born resulting in different participants in the follow-up occasions. This fact limits the evaluation of the results in this group of participants, and hampers the transferability.

The mixed method study included outcome values over time in both groups of women. The focus group interviews in the target minority group of women illustrates a persistent increased awareness of vitamin D and knowledge of lifestyle changes that improve its levels. It also highlights diverging opinions and feelings about obstacles for lifestyle and tradition changes. Vitamin D status is improved by consumption of vitamin D-rich foods, supplements and sun exposure of unveiled skin, which is particularly important for women at risk for ill health due to vitamin D insufficiency. Our study implies that cultural and linguistically adapted brief oral information provided by doctors would be especially efficient for these women at special risk.

## Conclusions

Brief oral information and illustrations about vitamin D given by doctors likely contributed to a change towards healthier food pattern and attitudes towards sun exposure, both in the short- and long-term among the Somali-born women with particularly low vitamin D levels and no prior knowledge. The effect of having a Somali nurse interpreter has not been evaluated here but could be of considerable importance in this process. Our preventive measures described here may have large impact on public health regarding bone, muscle and immunity, especially in particular risk groups for vitamin D deficiency.

## Data Availability

The datasets include sensitive and vital data that possibly could discredit a marginalised group of immigrant women in Sweden. Due to ethical reasons, datasets collected and/or analysed during the study are available from the corresponding author on reasonable request.
